# Computational Fluid Dynamics and Virtual Septoplasty in Nasal Airway Obstruction: A Narrative Review

**DOI:** 10.7759/cureus.101284

**Published:** 2026-01-11

**Authors:** Ian T Braithwaite, Amelia Jones, Claire Doherty, Dave K Sharma

**Affiliations:** 1 Otolaryngology, Royal London Hospital, Barts Health NHS Trust, London, GBR

**Keywords:** computational fluid dynamics, nasal airflow, nasal airway obstruction, septoplasty, virtual septoplasty

## Abstract

Nasal airway obstruction (NAO) is a common symptom with a substantial impact on quality of life. Septoplasty is frequently performed, yet outcomes remain variable and may decline over time. Computational fluid dynamics (CFD) enables patient-specific simulation of nasal airflow. Virtual septoplasty builds on this by allowing preoperative anatomical models to be digitally modified, with CFD used to estimate the functional impact of proposed surgical changes. This narrative review synthesises the contemporary CFD evidence base relevant to septoplasty and, to our knowledge, provides the first synthesis of the virtual septoplasty literature.

A focused narrative review was undertaken. Database searches up to November 2025 were screened for relevance. Structured data extraction was performed for eligible studies, followed by full-text review, independent assessment by two reviewers, and narrative synthesis.

CFD provides a detailed simulation of individualised nasal airflow but must be interpreted in the context of known limitations and modelling assumptions. Across studies, CFD metrics show stronger correlation with validated symptom scores than resistance measures, although reported correlations vary and are influenced by nasal cycle control and methodological heterogeneity. Virtual septoplasty studies demonstrate that digitally edited models can reproduce the direction and approximate magnitude of postoperative changes in airflow-related metrics, but evidence remains limited by small cohorts and limited comparison to validated symptom scores.

CFD and virtual septoplasty represent promising technologies for individualised assessment and surgical planning. Wider adoption will require standardised modelling workflows, validated symptom-based endpoints, and prospective evidence demonstrating improved outcomes over current clinical practice.

## Introduction and background

Nasal airway obstruction (NAO) is a common and heterogeneous condition, with structural, physiological, pathological, and sensory contributors. It affects up to one-third of the population [1] and has a substantial impact on daily functioning and quality of life [2].

Septoplasty is a commonly performed procedure to improve nasal airflow and reduce symptom burden, with reported success rates ranging from 27% to 84%, six months to 11 years after surgery, and studies suggesting that symptom relief often deteriorates over time [3-5] and a significant minority remain obstructed or dissatisfied after surgery. Given this variability in outcomes, there is a continued drive to optimise nasal airway surgery in terms of patient assessment, selection, and procedural planning.

The assessment of NAO is challenging. In routine practice, assessment combines clinical examination, validated symptom scores such as the Nasal Obstruction and Septoplasty Effectiveness (NOSE) score [6] and visual analogue scales (VAS) [7], and measurements of total nasal resistance/airway characteristics using techniques such as rhinomanometry, acoustic rhinometry, and nasal peak flow [8,9]. These objective measures demonstrate limited and variable correlation with patient-reported symptom scores and do not localise the anatomical source of obstruction [10].

Emerging methods use computational fluid dynamics (CFD) to simulate and quantify nasal airflow within patient-specific three-dimensional airway models. CFD offers the potential for a more objective and detailed assessment of nasal airflow [8,9,11], including localisation of functionally important sites of narrowing or altered flow [9,12,13]. These models can also be edited to perform virtual surgery, allowing surgeons to test different procedures in silico and predict their effects on airflow, resistance, and related metrics [14-16].

Existing reviews of CFD in rhinology have provided important overviews of CFD methodology and its role in assessing NAO [9,17]. Building on these, this narrative review takes a clinically oriented perspective focused on septoplasty. It offers, to our knowledge, the first focused synthesis of the emerging literature on virtual septoplasty and CFD-guided operative planning.

## Review

Methods

A focused narrative review of the literature on CFD, septoplasty, and virtual septoplasty was performed. A database search was conducted using PubMed/MEDLINE and Google Scholar up to November 2025. The initial search yielded 251 publications. Titles and abstracts were screened for relevance, with exclusion of non-clinical engineering studies, studies not addressing septoplasty or NAO, and non-human or non-English publications (n=138).

Following abstract screening, full-text assessment was undertaken for eligible articles. After full-text review, 54 papers were included as core clinical CFD studies, with a further 59 background papers retained to support interpretation and contextualisation. Additional relevant studies were identified during full-text review.

Structured data extraction was undertaken for all core studies, including study design, population, imaging and CFD workflow, principal CFD findings, clinical outcomes, and reported limitations. Two reviewers (IB and AJ) independently analysed each core paper, and findings were subsequently synthesised through iterative discussion. All symptom scoring instruments discussed in this review are non-proprietary tools that are freely available for clinical and research use. As this was a clinically oriented narrative review, a formal risk-of-bias assessment using standardised appraisal tools was not undertaken. Findings from individual studies are described qualitatively as reported by the original authors, and no re-analysis or pooling of statistical results was undertaken.

Given the scope of the review, the heterogeneity of study designs, CFD workflows, boundary conditions, outcome measures, and generally small to moderate sample sizes, meta-analysis was not performed. Instead, findings were synthesised narratively and organised into three domains: (1) CFD methods and limitations, (2) clinical and symptom correlation, and (3) virtual septoplasty and CFD-guided planning.

CFD in NAO

CFD is a field of applied physics and engineering that uses computers to solve the governing equations of fluid flow and predict how gases and liquids move. It allows airflow and heat exchange to be simulated in complex three-dimensional geometries, such as a model of an individual patient’s nasal airway.

CFD Process

To build such a model, a high-resolution CT scan is obtained, and the images are segmented to separate air from tissue based on CT radiodensity (Hounsfield unit) values. This segmentation is then refined with semi-automatic and manual editing and smoothed to create a three-dimensional airspace model that represents the patient's individual nasal airway [8,9,18,19].

The nasal airspace is then discretised into a computational mesh (a three-dimensional grid of small cells in which the flow equations are solved), and the mucosal walls are represented by surface elements that form the boundary of this mesh. Airflow is computed using either Navier-Stokes solvers on finite volume meshes or lattice Boltzmann methods on voxel-based grids [19-23]. Lattice Boltzmann approaches can exploit the native CT voxel structure (the regular three-dimensional array of CT volume pixels), which may improve meshing simplicity and computational efficiency [20,23].

Boundary Conditions

Boundary conditions define how air is driven through the model, usually by prescribing either a fixed transnasal pressure drop or a fixed inspiratory flow rate representative of resting nasal breathing, with simulations run as steady laminar flow [22,24-27]. Segalerba et al. compared constant pressure gradient, constant flow rate, and constant inlet pressure and showed that each produced different changes in calculated resistance and aerodynamic power for the same pre and postoperative geometries, emphasising the importance of boundary conditions as a differentiating factor in various CFD studies [19].

CFD Outputs

For clinicians, the main CFD outputs can be grouped into a small set of variables, typically reported either for the whole cavity or for anatomically defined segments such as the nasal valve, middle meatus, or regions around septal deviations [17,21,22,26]. These categories include airflow and flow partition, encompassing metrics such as unilateral and bilateral flow rates and the proportion of total airflow passing through each side or region; nasal resistance, most often expressed as pressure drop divided by flow (ΔP/Q), reported bilaterally or unilaterally for the whole nose or specific segments; and heat transfer, incorporating measures such as total mucosal heat flux and the surface area exceeding a defined heat flux threshold as surrogates for mucosal cooling. Additional categories include pressure and wall shear stress, typically represented by static or total pressure distributions and wall shear maps reflecting energy loss and friction along the mucosa, as well as velocity-based outputs, including velocity magnitude, direction, and streamlines, which are commonly used to visualise jets, recirculation, and low-flow zones [17].

Fluid Structure Interaction Limitations and the Nasal Valve

Most nasal CFD models assume rigid, nonmoving walls and a fixed mucosal temperature and do not incorporate fluid-structure interaction (FSI) at the nasal valve [9,22,24]. Modelling FSI is technically challenging because the mechanical properties and motion of the nasal sidewalls are difficult to measure in vivo, so current simulations generally treat the nose as a rigid conduit [9]. Exclusive nasal breathing usually occurs during resting respiration at relatively low flow rates, and inward inspiratory shifting of the anterior nasal sidewalls is typically small under these conditions, so neglecting wall motion may introduce only minor error for resting flow states but rigid wall CFD still cannot capture dynamic or asymmetric nasal valve behaviour, which may become important at higher inspiratory flows and in patients with valve weakness or marked side-to-side differences [9], underscoring the importance of clinical assessment for nasal valve pathology.

Confounding Effect of the Nasal Cycle

The nasal cycle is a further constraint for nasal CFD and can cause up to a fivefold change in unilateral airflow [25]. In vivo measurements suggest that transitions between cycling states are relatively rapid, so most of the time the nose sits near a congested or a decongested plateau [28]. Different strategies have been used to handle this. Some studies decongest patients before imaging to reduce cycle-related asymmetry [29]. Others exclude patients with clear side-to-side mucosal differences or obvious cycling on serial scans, which can remove a substantial proportion of eligible cases and introduce selection bias [13,21,24,27].

Groups have also created "extreme" and "mid-cycle" models by using CT scans in opposite congestion states and then morphing turbinate and septal swell body thickness to generate an intermediate, mid-cycle geometry [25,26]. These approaches can prevent misleading pre- versus postoperative comparisons in which nasal cycle shifts dominate over surgical change and can strengthen correlations between CFD variables and subjective patency.

More recently, image-based morphing approaches have been proposed to address nasal cycle confounding without reliance on serial imaging. Using active-contour and path-planning algorithms, Vithanage et al. generated predicted intermediate turbinate geometries between congested and decongested states from a single scan and validated these against serial scans, demonstrating reasonable accuracy [30]. While currently in small datasets, such methods offer a promising technique for generating CFD models from a single preoperative scan which account for nasal cycle variation. This could improve symptom correlation and support future applications in surgical planning without serial scans and associated radiation exposure.

Taken together, CFD provides a detailed, patient-specific map of airflow, resistance, and mucosal cooling that goes beyond traditional global measures, but important assumptions and limitations must be considered when judging its usability for clinical assessment, operative planning, and virtual septoplasty. These methodological considerations provide important context for interpreting which CFD-derived variables are most relevant to patient experience, as explored in the following section.

Clinical correlation between CFD and patient-reported outcomes

Classical objective tests such as rhinomanometry and acoustic rhinometry show only modest and inconsistent correlations with perceived nasal obstruction [9,12,17]. CFD can instead characterise airflow and heat transfer in a patient-specific, side-specific, and region-specific manner, raising the possibility that model-based metrics could align more closely with an individual's perception of nasal airflow. Across the NAO literature, NOSE and VAS scores are the most widely used and validated patient-reported outcome measures, although some CFD series have used non-standard perception scales [17].

The mechanism responsible for nasal airflow sensation remains incompletely understood. Multiple studies have shown that subjective nasal patency correlates poorly with nasal resistance measured by rhinomanometry, supporting the concept that patients present because of a subjective perception of decreased patency rather than a simple increase in resistance [12]. Resistance is clearly related to airflow, but appears to be a distinct variable from perceived patency, which recent experimental and CFD work suggests is more closely linked to patterns of mucosal cooling and local mechanical stimulation of the mucosa, rather than total nasal resistance alone [9,17,21,24].

Limitations of Nasal Resistance

As suggested, nasal resistance shows a less consistent relationship with patient-reported outcomes than might be expected. Casey et al. found higher nasal resistance in NAO patients than in healthy controls but no correlation between nasal resistance and NOSE or VAS [12]. Gaberino et al. reported that correlations between nasal resistance and NOSE/VAS emerged only after the virtual correction of the nasal cycle [26]. Complicating matters, there is definitional variability between papers, with nasal resistance calculated between different intranasal landmarks; Kim et al. found stronger correlations with symptoms when nasal resistance was measured from the naris to the end of the septum rather than from the naris to the nasopharynx [31].

Laterality and Affected Side Metrics

CFD studies consistently suggest that metrics on the clinically obstructed side correlate better with symptoms than global bilateral resistance. In two small pre- and postoperative cohorts, Kimbell et al. showed that unilateral CFD-derived nasal resistance and heat transfer on the more obstructed side tracked symptom improvement, whereas bilateral resistance did not; patients with the largest reduction in affected side resistance and increased mucosal heat transfer reported the greatest relief even when total bilateral resistance changed little [21,22].

Chiang and Frank-Ito, in a postoperative cohort, identified a model in which postoperative VAS on the initially affected side and postoperative CFD-derived airflow in that side explained much of the variation in postoperative NOSE, suggesting that outcome perception is driven by the postoperative state of the original affected side rather than by bilateral averages [13].

Side Asymmetry and Obstruction Phenotypes

Side-to-side asymmetry in airflow and pressure appears closely linked to symptom burden. In a cohort of 232 patients with septal deviation, Janović et al. demonstrated that Mladina types 1, 2, 4, and 7, in which the deviation involves the internal nasal valve region, exhibited the greatest asymmetry in CFD-derived resistance and pressure between sides and the highest NOSE scores. Across deviation types, the magnitude of asymmetric resistance difference correlated strongly with mean NOSE score, supporting resistance asymmetry and valve-level obstruction as key determinants of symptom severity [29].

These findings are consistent with work showing that subjective nasal patency is more closely related to regional and asymmetric airflow patterns than to global resistance. Studies examining airflow distribution have shown that reduced flow through the middle airway and preferential routing of airflow away from this region are associated with worse patency perception [12]. This also echoes earlier rhinomanometry and CFD studies demonstrating that anterior septal deviations markedly increase nasal resistance, whereas more posterior deviations may have little effect on overall resistance [32].

Using combined CFD and clinical data, Barbarite et al. further characterised obstruction phenotypes and showed that in certain patterns, particularly unilateral or dynamic disease, global resistance was a poor surrogate for symptoms. Instead, disturbed airflow at the nasal valve and within the middle airway, with locally elevated resistance and shear around the deviation and anterior turbinates, better explained persistent obstruction despite adequate inferior airway calibre [8].

Mucosal Cooling, Heat Flux, and Symptom Perception

Several CFD studies suggest that mucosal cooling is a more sensitive correlate of nasal patency sensation than resistance alone. Sullivan et al. showed that, in patients undergoing surgery for nasal obstruction, the surface area of the more obstructed side cavity with heat flux above 50 W/m² had the strongest correlation with NOSE score and VAS, outperforming peak heat flux and total heat transfer; patients who felt better after surgery had a clear increase in this high heat flux area, particularly in the middle and posterior parts of the nasal cavity [24].

In a cohort with septal deviation, Radulesco et al. reported that regional heat flux measured 1 cm posterior to maximum deviation on the obstructed side showed a very strong correlation with their perception score, whereas more global CFD variables were less discriminating [33]. Na et al. similarly found that improvement in NOSE score after septoturbinoplasty was linked not only to a fall in unilateral resistance in the more obstructed side but also to an increase in heat flux between the nasal valve and choana [27].

Some studies have suggested that the importance of mucosal cooling and middle airflow might be explained by an uneven distribution of sensory receptors within the nasal cavity. However, although sensory receptors are heterogeneously distributed within the nasal mucosa, menthol sensation appears relatively uniform throughout the cavity [34] and is closely related to mucosal cooling mediated by the cold/menthol receptor TRPM8, which has been confirmed in nasal mucosal biopsies [35]. Casey et al. therefore argued that it is the pattern of mucosal cooling generated by airflow, particularly in the middle airway, rather than receptor distribution itself, that drives patency perception; in their cohort, middle airflow on the narrow side correlated most strongly with NOSE and VAS scores and was closely linked to heat flux [12].

Wall Shear Stress

Wall shear stress reflects the frictional force exerted by airflow on the mucosal surface and is thought to be linked to both mechano- and thermoreceptor stimulation [9]. Kimbell et al. reported that wall shear stress on the affected side was strongly associated with NOSE score [22]. There are, however, important limitations to wall shear stress as a standalone metric. Wall shear stress falls to zero in patients with complete obstruction [17], and it can be misleading after inferior turbinate reduction; in a series of patients with empty nose syndrome, Li et al. noted paradoxically low inferior wall shear stress [36,37]. With these caveats in mind, wall shear stress can still provide useful information about a patient's obstruction, particularly when interpreted alongside regional heat flux.

Despite these coherent patterns, the evidence base for CFD and symptom correlation remains early. Most studies use small, selected cohorts, often with exclusions for imaging quality and nasal cycle control. There is methodological variation in segmentation, meshing, and boundary conditions [17,21,26,27], and several CFD series report only flow and resistance-based metrics without incorporating patient-reported outcome measures, so the link between model outputs and patient experience is indirect in much of the literature. Overall, current data suggest that affected side, asymmetric, and regional CFD variables, particularly those capturing mucosal cooling and flow distribution around the deviation and valve, are the most promising correlates of nasal obstruction symptoms, but they still need to be interpreted alongside full clinical assessment, and larger longitudinal studies will be required before CFD can be integrated into routine septoplasty planning and outcome evaluation.

Virtual septoplasty and CFD-guided planning

Virtual septoplasty involves digitally modifying a patient's nasal airway anatomy to reflect a proposed surgical change, running CFD on the edited model, and interpreting the resulting changes in airflow, resistance, and heat transfer as a proxy for surgical effect.

Procedural Planning

In NAO surgery, there is a clear aim to identify which surgical components provide the greatest functional gain while limiting operative extent and risk. Rhee et al. addressed this by manually editing preoperative CT-based airway models to mimic the geometric effect of different surgical procedures, generating virtual postoperative anatomies, and running CFD on each to compare predicted nasal resistance and airflow patterns [38,39].

Across an index case and a subsequent series, they created virtual models representing septoplasty alone, inferior turbinate reduction alone, combined septoplasty plus turbinate surgery, and, in later work, additional nasal valve procedures; septoplasty and valve-level corrections accounted for most of the predicted reduction in resistance and improvement in flow symmetry, turbinate reduction alone had little effect, and adding turbinate surgery to septoplasty provided only modest additional benefit [38,39].

Frank-Ito et al. generalised this concept into a stepwise planning framework, applying sequential virtual edits to the preoperative model and using CFD to estimate the marginal gain of each added surgical step [15]. This work illustrates how virtual septoplasty can be used prospectively to compare alternative strategies for a given patient and to predict surgical steps that produce the greatest functional benefit.

Burgos et al. examined how such tools interact with real surgical decision-making by asking six surgeons to plan surgery on the same anatomy within a three-dimensional virtual surgery platform [40]. Initial plans showed substantial variation in the type and extent of septal and turbinate work and produced differing predicted patterns of resistance and wall shear stress. After surgeons were shown CFD models of their own and alternative plans, many modified their intended procedures in light of the predicted airflow changes [40].

Comparison With Postoperative CFD

Rhee et al. were the first to compare virtual surgery CFD with CFD from actual postoperative anatomy. Using the preoperative CT, they created virtual models of septoplasty, inferior turbinate reduction, and septoplasty plus inferior turbinate reduction; the patient later underwent septoplasty with right inferior turbinate reduction. Six-month postoperative CFD most closely matched the septoplasty plus inferior turbinate virtual model but showed larger reductions in resistance and greater flow symmetry, indicating that virtual surgery predicted the pattern of change while slightly underestimating the magnitude of effect [38].

In a cohort of 10 patients who underwent various combinations of septoplasty, septorhinoplasty, turbinectomy, and nasal valve procedures, Frank-Ito et al. hand-edited the preoperative CT models after surgery to create "transcribed-surgery" geometries intended to reproduce the operations actually performed. CFD outputs from both the transcribed and true postoperative models showed the expected reductions in resistance and increases in airflow on the predominately obstructed side (POS) compared with the preoperative models; however, the transcribed models tended to overestimate improvements in bilateral resistance, which the authors attributed to coarse two-dimensional editing, nasal cycle effects, and unmodelled healing [41].

Expanding validation beyond resistance and airflow, Radulesco et al. evaluated a wider set of CFD variables in two virtual septoplasty cases. Editing preoperative CT airspaces to simulate realistic resections, they found that virtual and actual surgery produced similar changes in mucosal heat flux, wall shear stress, pressure, temperature, and CFD-derived resistance, with virtual models slightly overestimating improvement [16]. Using the DigBody platform, Burgos et al. likewise showed that postoperative airflow, pressure, and heat transfer metrics closely matched those predicted by preoperative virtual surgery [18].

Overall, these small comparisons suggest that carefully constructed virtual septoplasty can reproduce the pattern and approximate size of postoperative changes in key CFD metrics, yet current implementations remain research tools that depend on both pre- and postoperative CT, tend towards overestimating benefit, and have limited validation against clinical or long-term outcomes.

Limitations of Early Manual Platforms and Shift to Algorithmic Methods

Early virtual surgery systems required labour-intensive, slice-by-slice editing of CT-derived airway models, making them operator dependent and poorly suited to realistic three-dimensional resections. Frank-Ito et al. highlighted the limitations of this two-dimensional editing, including difficulty reproducing true three-dimensional changes and the absence of any modelling of healing [41]. Clipp et al.'s near-real-time lattice Boltzmann planner improved interactivity but remained restricted by limited editing tools and a complex interface [23]. Later three-dimensional sculpting platforms were more intuitive but still relied on detailed manual editing and were best suited to simple resections [18]. These constraints, combined with broader CFD and segmentation variability, have motivated the shift toward more standardised and automated virtual septoplasty methods [9].

Atlas-based methods use a "healthy" template nose (statistical shape model (SSM)) to guide semi-automated septal corrections. Moghaddam et al. built an atlas from healthy CTs, mapped regions of abnormal septal deviation, and applied local corrections toward the atlas geometry; in a proof-of-concept case, the resulting virtual septoplasty produced CFD resistance and airflow patterns similar to postoperative findings [42]. Vicory et al. used a related approach in a larger cohort, automatically generating straightening plans, but several were judged over-straightened or unrealistic [43], showing that atlas-driven automation requires caution to achieve surgically plausible outcomes. These methods also risk bias if the atlas is drawn from an unrepresentative population.

Reinforcement learning (an artificial intelligence (AI) method in which an agent tries actions, receives rewards, and learns the best strategy) offers a further layer of automation. Rüttgers et al. integrated this framework with CFD, allowing an agent to propose local septal and turbinate edits, evaluate each with CFD, and iteratively refine its approach to maximise a reward based on resistance and thermal metrics [44]. In test scenarios, the reward-optimised plans tended toward overly aggressive straightening and turbinate reduction, illustrating that without anatomical and clinical constraints, automated optimisation may generate surgically unrealistic solutions.

Technological Adoption

Few studies have examined how surgeons might integrate virtual septoplasty into routine practice. In a study by Vanhille et al., nine otolaryngologists reviewed pre-generated virtual surgery models and corresponding CFD outputs for a real NAO case. Surgeons rated the models as moderately realistic (mean 3.4/5) and reported improved understanding of functional consequences and enhanced confidence in counselling, but only modest influence on operative choice (mean 2.6/5). Perceived usefulness was greatest for patient counselling, candidate selection, and decisions around turbinate surgery, while time requirements, CT radiation, and the absence of real-time editing were identified as key barriers [11]. Burgos et al. similarly reported that three-dimensional virtual surgery environments were valuable for explaining proposed resections to patients and trainees, despite limited predictive functionality [18].

Overall, these early adoption studies suggest that surgeons view virtual septoplasty as a useful adjunct for understanding and communicating functional effects of surgery, and in selected cases for refining the extent and targets of surgery, but that current platforms remain research prototypes constrained by CT-based workflows, modelling overhead, and limited prospective validation against patient-reported outcomes.

## Conclusions

CFD provides a detailed, patient-specific characterisation of nasal airflow. However, important methodological and technical limitations must be considered. Current workflows depend on modelling assumptions and implementation choices, are constrained by nasal cycle effects, and have limited ability to capture dynamic or asymmetric nasal valve behaviour. CFD outputs, therefore, need to be interpreted alongside full clinical assessment, and further methodological standardisation is required, particularly in ongoing development of approaches to manage nasal cycle effects without relying on serial or postoperative imaging. Across clinical studies, several CFD variables show closer alignment with symptom experience than classical resistance-based measures. Larger longitudinal studies using standardised workflows and validated symptom scoring are needed to confirm these patterns and define clinically meaningful thresholds.

Virtual septoplasty uses CFD to support surgical planning and patient counselling by enabling the preoperative evaluation of proposed procedures. Early studies show that well-constructed virtual edits can approximate postoperative changes in airflow, resistance, and heat flux and may help identify functionally important targets. However, evidence remains limited: studies are small, rely on labour-intensive workflows, seldom include validated symptom scores, and tend to overestimate benefit. Newer sculpting, atlas-based, and reinforcement learning approaches aim to improve speed and automation but remain at an early stage and must incorporate appropriate clinical constraints and minimise bias. CFD and virtual septoplasty nonetheless represent promising technological developments with the potential to individualise assessment and refine surgical planning. Realising this potential will require streamlined, standardised workflows, consensus on clinically meaningful CFD endpoints, validated symptom-based outcomes, and prospective evidence that CFD-guided planning improves long-term outcomes above current standards of care.
